# Study on the Material Removal Mechanism of FGH99 by Laser-Induced Microjet Assisted Ablation at Different Incidence Angles

**DOI:** 10.3390/mi17040475

**Published:** 2026-04-15

**Authors:** Yixin Duan, Zhen Zhang, Zefei Zhu, Jing Ni

**Affiliations:** School of Mechanical Engineering, Hangzhou Dianzi University, Hangzhou 310018, China; 231010003@hdu.edu.cn (Y.D.);

**Keywords:** laser ablation, laser-induced microjet, FGH99 superalloy, surface microstructure, material removal mechanism

## Abstract

Laser-induced microjet-assisted ablation is an emerging technology in the field of laser processing. However, the influence of solid boundaries on jet behavior and the associated material removal mechanism remains unclear after observing and analyzing the ablation process. To address this, the present study systematically investigates the effect of the incidence angle on the processing efficiency and material removal mechanism in laser-induced microjet ablation. By controlling the laser power and liquid layer thickness, the dynamic behavior of the microjet, material removal performance, and surface morphology evolution under different inclination angles were explored. Based on video analysis and OpenCV processing, the regulation of jet morphology and impact mode by the incidence angle was revealed. Combined with white light interferometry and ultra-depth-of-field three-dimensional microscopy, the ablation depth and material removal rate were quantitatively characterized. The results showed that under normal incidence, the maximum material removal rate of 0.092 mm^3^/s was achieved at 9 W, while further increases in power led to a decrease in removal rate due to bubble aggregation. When the sample was tilted to 15°, the material removal rate reached 0.163 mm^3^/s, representing a 106.30% improvement compared to that at 0°, and the ablation depth also peaked with an average maximum depth of 12.32 ± 0.58 μm and a single-point maximum of 54.36 μm. Furthermore, scanning electron microscopy (SEM) and energy-dispersive X-ray spectroscopy (EDS) were employed to elucidate the microstructural features and elemental distribution under different process parameters. Through multi-parameter experiments, this study achieved process parameter optimization and clarified the material removal mechanism influenced by different incidence angles, providing both a process reference and theoretical basis for efficient micro-machining of aerospace materials.

## 1. Introduction

Nickel-based superalloy FGH99, as the third-generation nickel-based powder metallurgy superalloy independently developed in China, is widely used in critical components of aeroengines [1], such as turbine disks and blades, due to its excellent high-temperature oxidation resistance, high toughness, high yield strength, and corrosion resistance. With the rapid advancement of the aerospace industry, the demands on material performance have become increasingly stringent. The enhancement of material properties is often accompanied by the need for surface microstructuring [2] or material removal [3]. Consequently, traditional machining methods [4,5] inevitably face certain limitations when processing such difficult-to-machine materials. Laser processing, particularly laser ablation [6], as a novel non-contact machining technique, enables the target material to be vaporized and removed within an extremely short time, thereby achieving processing objectives. It is recognized as an efficient and precise machining method.

Laser ablation conducted in air is referred to as direct laser ablation [7]. This process can lead to reduced machining quality due to thermal effects, resulting in issues such as cracks [8], recast layers [9], and ablation debris deposition [10]. Although laser sources have evolved from nanosecond and picosecond [11] to femtosecond [12] regimes, transitioning from long pulses to ultrafast short pulses, picosecond and femtosecond lasers remain costly and cannot completely eliminate heat accumulation effects during the ablation process. Therefore, a significant body of research has shifted towards laser ablation in liquid environments, utilizing liquids with high thermal conductivity to effectively suppress the thermal effects of laser ablation [13,14,15,16]. Water is commonly used due to its high specific heat capacity and favorable thermal stability. Building on this, studies have investigated the coupling relationships between laser parameters and variables such as water layer thickness [17], flow dynamics [18], and temperature [19], or have employed energy field assistance to enhance the laser ablation process [20].

Laser-induced microjets were initially applied in the field of medical injection [21]. Subsequently, in continuous laser ablation processing, the presence of a solid boundary was found to cause the asymmetric collapse of induced cavitation bubbles, thereby generating directional microjets. This characteristic has gradually been introduced into the manufacturing sector, leading to the development of laser-induced microjet-assisted laser ablation technology [22]. However, the influence of changes in the solid boundary, such as the sample inclination angle, on jet behavior and overall processing outcomes has not yet been systematically investigated.

Therefore, based on this research gap, this paper first investigates the processing characteristics and surface morphology under normal incidence at different laser powers. Subsequently, by varying the sample inclination angle, the study explores the regulatory role of the solid boundary on cavitation bubble dynamics and analyzes the results of laser-induced microjet-assisted ablation processing at different angles. Finally, the influence of the incidence angle on the material removal mechanism of laser-induced microjet-assisted ablation is elucidated from three perspectives: laser incidence angle, liquid layer perturbation, and jet-induced pressure.

## 2. Materials and Experimental Setup

### 2.1. Material and Sample Preparation

The material used in this experiment was FGH99 alloy (Mida Machinery Technology Co., Ltd., Wuhu, China), with dimensions of 15 mm × 5 mm × 2 mm. The elemental composition of the FGH99 alloy is shown in Table 1. Prior to laser ablation, the machined surface was polished using sandpapers of various grit sizes (400, 600, 800, 1000, 1200, and 1500). The polished sample was then ultrasonically cleaned in ethanol for 10 min and subsequently dried. After treatment, the surface roughness (Ra) of the sample was approximately 0.2 μm.

### 2.2. Laser Ablation Experiment

**Figure 1:**
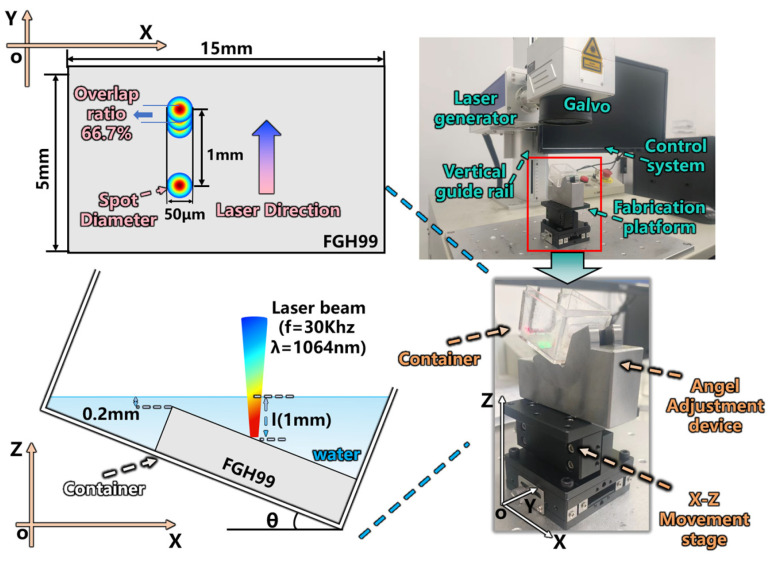
Schematic diagram of the machining process.

A pulsed laser system (model H20, Han’s Laser Technology Industry Group Co., Ltd., Shenzhen, China) with a wavelength of 1064 nm was employed. The maximum output power of the laser was 30 W, and the pulse frequency was adjustable within the range of 10–1000 kHz. The detailed experimental parameters for the laser ablation process are summarized in Table 2. The sample was first fixed at the bottom of a container, which was adjusted to a predefined inclination angle *θ* using an adjustable angle gauge and placed on an X-Z motion control stage with a positioning accuracy of 5 μm. A reference line was marked on the outer wall of the container at 0.2 mm above the sample surface using a height gauge. The laser beam was pre-aligned to the upper edge of the sample as the starting point. Based on the cosine of the target angle *θ*, the motion stage was employed to displace the sample by a calculated distance along the X-direction, thereby positioning the pre-aligned laser spot exactly 1 mm beneath the liquid surface. Subsequently, the liquid was injected into the container, and its thickness was precisely adjusted to the target value using a micropipette. Finally, linear ablation was performed on the pre-aligned region over a scanning length of 1 mm with 500 repeated passes. A schematic illustration of the experimental setup is shown in Figure 1. To ensure the reliability and reproducibility of the experimental results, each set of experimental parameters was repeatedly machined and analyzed using at least three independent machining grooves.

A motion camera (S5KGW1, Samsung Electronics Co., Ltd., Suwon, Republic of Korea) (4K, 30 fps) was employed to record the ablation process, and the acquired images were analyzed using OpenCV (version 4.5.2). Using OpenCV, the displacement of the jet front between different frames is tracked. After the jet morphology stabilizes, adjacent frames are selected to calculate the distance traveled by the jet front. This distance is then divided by the frame interval (0.5 s), and the average value is taken as the jet velocity under the given condition. An ultra-depth-of-field microscope (KEYENCE VW-9000, Osaka, Japan) was used to observe the surface morphology of the ablated regions. A white light interferometer (Sensofar S neox 3D, Terrassa, Spain) was utilized to characterize the average geometric profile of the machined grooves and to measure the average maximum ablation depth and material removal rate. A scanning electron microscope (JSM-IT700HR/LA, JEOL Ltd., Tokyo, Japan) was employed to capture the micro-morphology of the ablated surfaces and to perform elemental composition analysis (EDS).

## 3. Results

### 3.1. Influence of Power on Jet Behavior and Macroscopic Surface Morphology at 0° Incidence

#### 3.1.1. OpenCV Program Design

**Figure 2:**
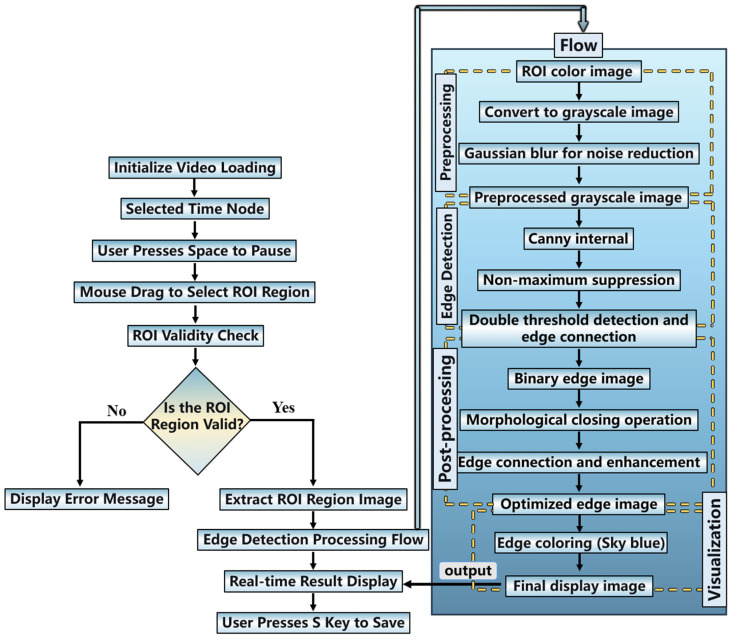
Flowchart of the OpenCV program logic.

In most cases, water-assisted laser ablation is commonly employed under normal incidence (0°). Therefore, in this study, the cavitation bubbles and jet morphology at different time nodes under this condition were first analyzed. A motion camera (4K, 30 fps) was used to record the processing at different laser powers, with images captured every 0.5 s. Meanwhile, the OpenCV image processing tool and the Canny edge detection algorithm were utilized to identify and process bubble features at nodes with distinct characteristics. The logical framework of the program is shown in Figure 2.

Upon system startup, the video file is first loaded to obtain basic information such as frame rate and resolution. Subsequently, three display windows are created: the main video window, the ROI edge detection result window, and the parameter adjustment window. The user can control video playback (pause/resume) using the spacebar, with region of interest (ROI) selection permitted only in the paused state. Once the ROI is selected, the system records its coordinates and marks it as selected. Thereafter, edge detection is performed on the ROI for every subsequent frame, regardless of the playback state.

The detection process begins with ROI extraction. The system first ensures that the ROI coordinates are within the video frame boundaries. If valid, the ROI sub-image is cropped from the current frame. The color ROI image is then converted to grayscale to simplify subsequent processing and reduce computational load. To suppress noise potentially introduced during image acquisition, a Gaussian blur with a kernel size of 3 × 3 is applied to the grayscale image, smoothing the image to prevent noise from being misidentified as edges.

The preprocessed image then enters the core edge detection stage, which employs the Canny algorithm. The Canny edge detection process consists of several sub-steps: first, the Sobel operator is used to compute the image gradient magnitude and direction; next, non-maximum suppression is applied to thin the edges, retaining only pixels with local gradient maxima; finally, double threshold detection is performed, classifying pixels into strong edges, weak edges, and non-edges. Edge connectivity is then used to retain weak edges connected to strong edges as the final edges (ensuring kernel sizes are odd to avoid computational errors). The binary edge image output by the Canny detector subsequently undergoes morphological post-processing, specifically a closing operation using a 2 × 2 rectangular structuring element. Closing, which consists of dilation followed by erosion, fills small gaps in the edges, connects nearby edge segments, and preserves the overall edge shape, resulting in a more continuous and complete detection.

To visually present the detection results, the system converts the binary edge image into a three-channel color image and sets all edge pixels to sky blue via a masking operation. This color edge image is then overlaid onto the original color ROI image with 30% transparency, generating a fused image that retains the original scene information while highlighting the edge locations. Simultaneously, the system calculates the total number of edge pixels and their proportion relative to the total ROI pixels (edge density). This statistical information is displayed in sky blue text at the top of the result image, facilitating quantitative assessment by the user.

Finally, the processed ROI image is displayed in real-time in the “ROI Edge Detection” window. The main video window also shows a thumbnail in the upper right corner of the video frame, indicating the current ROI box, edge pixel count, detection parameters, and operation prompts. We can press the ‘S’ key to save the current ROI detection result as a PNG file for subsequent analysis.

#### 3.1.2. Jet Evolution Under Different Laser Powers

**Figure 3:**
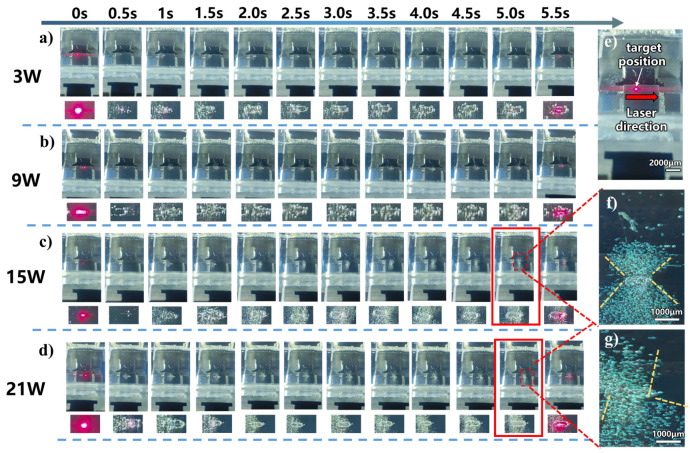
Jet evolution at different laser powers and a liquid thickness of 1 mm: (**a**) 3 W, (**b**) 9 W, (**c**) 15 W, (**d**) 21 W, (**e**) processing example, (**f**) OpenCV processed image (15 W 5.0 s), (**g**) OpenCV processed image (21 W 5.0 s).

Frames were captured from the processing videos at 0.5 s intervals for different laser powers, and specific feature points were characterized using the aforementioned code. The characterization results are shown in Figure 3.

As the laser power increases, the jet becomes progressively more pronounced. Under low-power conditions (3 W, 9 W), a stable jet fails to form. The generated cavitation bubbles either diffuse randomly or accumulate and merge, ultimately adhering to the periphery of the machined groove (Figure 3a,b). This phenomenon can be attributed to insufficient laser energy, which can barely induce bubble generation but is inadequate to support violent bubble collapse. Concurrently, the Marangoni effect exacerbates this behavior: the laser ablation process creates a significant local temperature gradient, with the highest temperature at the groove bottom (the direct laser interaction zone) and relatively lower temperatures at the groove walls and surrounding liquid. This temperature gradient induces a surface tension gradient—lower in the high-temperature region and higher in the low-temperature region—generating a thermocapillary force directed toward the high-temperature zone. This force drives the bubbles to migrate toward and adhere to the groove walls.

At 15 W, the jet evolves from initial disordered bubble disturbances into a stable, well-defined morphology. Image processing using OpenCV at t_1_ = 5.0 s reveals a vertically symmetric jet with a specific divergence angle (Figure 3c,e). This evolution can be explained as follows: in the initial stage, stochastic bubble nucleation induced by laser irradiation leads to chaotic bubble behavior. As processing continues, heat accumulation raises the melt pool temperature, and the periodic laser pulses gradually regularize bubble dynamics. A stable high-temperature, high-pressure core region is established at the melt pool center, enabling relatively symmetric bubble collapse. The resulting jet, influenced by the resistance of the liquid medium, propagates outward with a conical diffusion morphology.

**Figure 4:**
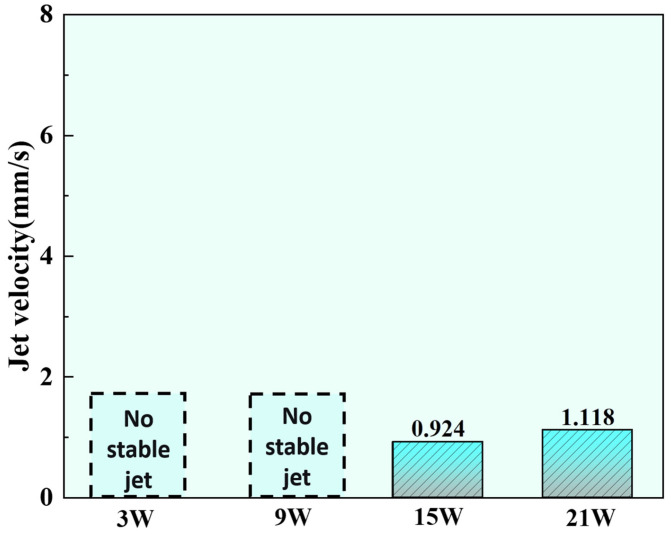
Diagram of jet velocity at different power under 0° and 1 mm.

When the power is further increased to 21 W, the jet evolution trend resembles that at 15 W. However, the intensified heat accumulation significantly elevates the processing zone temperature, lowering the cavitation threshold and dramatically increasing the number of bubbles generated per pulse. Simultaneously, the enhanced Marangoni effect at higher temperatures promotes more pronounced bubble migration toward the walls and subsequent coalescence. Although the jet velocity increases slightly with power, from 0.924 mm/s at 15 W to 1.118 mm/s at 21 W (Figure 4), its expulsion capacity is limited, preventing timely removal of the excessive bubbles. These retained bubbles coalesce and spread at the tail of the processing zone, forming local accumulations that disrupt flow field stability. Consequently, the jet loses the symmetry and directionality observed at 15 W (Figure 3d,f).

#### 3.1.3. Effect of Different Laser Powers on Ablation Morphology and Resultant Surface Characteristics

**Figure 5:**
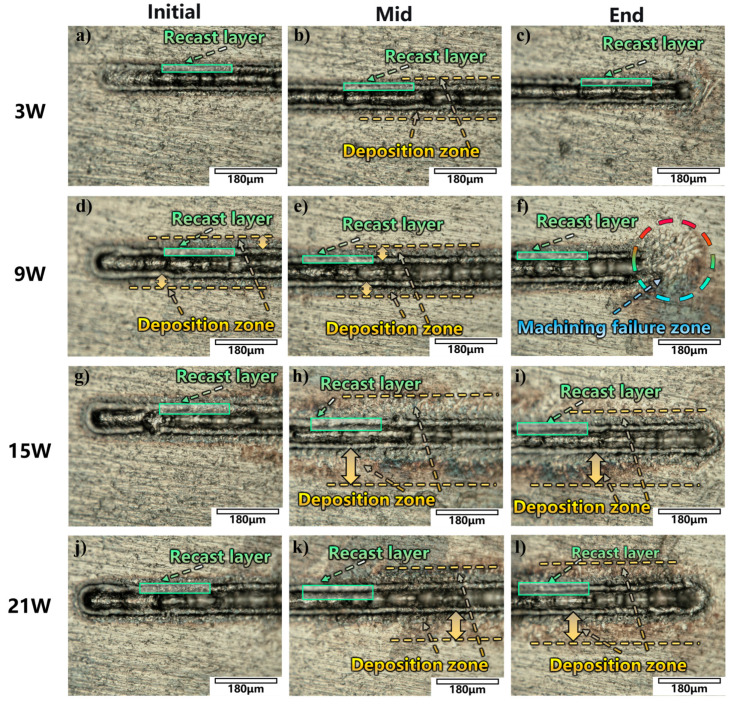
Macroscopic morphologies of the machined surface at different positions under various laser powers with a liquid thickness of 1 mm: (**a**–**c**) 3 W, (**d**–**f**) 9 W, (**g**–**i**) 15 W, (**j**–**l**) 21 W.

After observing and analyzing the ablation process under different power levels, the surface morphology of the machined samples was examined using an ultra-depth-of-field microscope. The machining results were shown in Figure 5.

At 9 W, an incompletely ablated region with debris splashing is observed at the tail of the processed groove (Figure 5f). To better verify this phenomenon, the experiment is repeated twice under the same conditions, and partially unformed machining regions are observed at the tail of the machining groove in both cases. Compared to 3 W, the number of cavitation bubbles increases at 9 W, but a directional and stable jet fails to form. This leads to bubble accumulation, coalescence, and in situ collapse at the tail region. The aggregated bubbles create a “barrier” that severely interferes with the laser transmission path, preventing the laser from effectively reaching the target surface and resulting in insufficient material removal.

When the power is increased to 15 W, a large material deposition zone appears in the central region of the processed area (Figure 5h). Ablation debris is distributed in a splashing pattern along the upper and lower edges of the groove, accompanied by a brownish oxidized surface. Based on the previous analysis of jet behavior at 15 W, although a relatively distinct jet is formed, its influence range is limited. Consequently, a substantial number of cavitation bubbles remain in the processing zone without being promptly expelled. These bubbles collapse in situ at the periphery of the jet-affected region, dissipating energy uniformly and ejecting molten debris onto both sides of the groove, forming deposition bands. Simultaneously, bubble coalescence occurs, and the resulting larger bubbles, upon collapse, can breach the liquid layer and come into contact with oxygen in the air, forming brownish oxides. Similar phenomena are also observed at the tail of the 15 W processed groove and in both the central and tail regions at 21 W (Figure 5i,k,l). These observations indicate that the contradiction between cavitation bubble dynamics and jet-induced debris removal capacity at high powers is a common cause of splashing deposition and surface damage.

**Figure 6:**
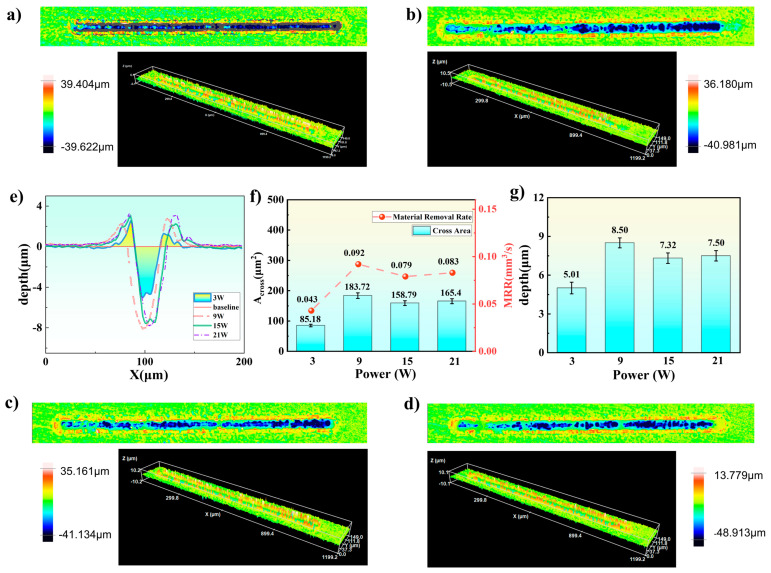
White light interferometry morphologies of ablation results under different laser powers: (**a**) 3 W, (**b**) 9 W, (**c**) 15 W, (**d**) 21 W, (**e**) average cross-sectional ablation profiles, (**f**) material removal rate and Groove cross-sectional area, (**g**) average maximum ablation depth.

Based on the preliminary observation of the machined surface morphology using an ultra-depth-of-field microscope, important information regarding the macroscopic profile of the ablation region, debris distribution, and oxidation characteristics was obtained. However, to further quantitatively evaluate the processing effects under different angles, a white light interferometer was employed to characterize the three-dimensional morphology of the ablated region. The ablation depth and material removal rate were quantitatively calculated, and the results were shown in Figure 6.

Under four different laser powers (3 W, 9 W, 15 W, and 21 W), the machined grooves exhibit distinct and continuous morphological characteristics, except for partial missing areas at 9 W, as shown in Figure 6a–d. The maximum single-point ablation depth, reaching 48.91 μm, is observed at 21 W (Figure 6d).

The average cross-sectional profiles (Figure 6e) reveal that the groove cross-sections are highly similar across different powers. However, an upward rebound phenomenon is observed at the bottom of the cross-sectional profiles at 3 W and 15 W. Notably, the average maximum ablation depth does not occur at the center of the cross-section, deviating from the central peak expected from the Gaussian beam energy distribution. This can be attributed to the stochastic nature of cavitation bubble nucleation: some bubbles form and remain in the processing zone during laser irradiation, exerting shielding and scattering effects on subsequent incident laser pulses. This alters the actual energy deposition distribution, resulting in non-centrosymmetric ablation cross-sections.

Further analysis of the effect of power on ablation depth shows that when the power increases from 3 W to 9 W, the cross-sectional area expands significantly, and the average maximum ablation depth increases from 5.01 ± 0.45 μm to 8.50 ± 0.39 μm—a substantial improvement of 69.7%. However, when the power is further increased to 15 W and 21 W, the ablation cross-section does not continue to expand; instead, it decreases slightly, with the average maximum ablation depth dropping to 7.32 ± 0.41 μm and 7.50 ± 0.40 μm, respectively (Figure 6f). Statistical analysis (*t*-test) reveals that the ablation depth at 9 W is significantly greater than those at 3 W (t = 10.22, *p* < 0.05), 15 W (t = 3.67, *p* < 0.05) and 21 W (t = 3.13, *p* < 0.05).

This phenomenon can be explained as follows: as the power increases, the number of laser-induced cavitation bubbles increases dramatically. However, the bubble removal capacity is limited by the finite jet-induced debris removal efficiency, leading to substantial bubble accumulation in the processing zone. These accumulated bubbles not only strongly scatter and absorb subsequent laser pulses but also impede effective energy transfer to the material surface, resulting in significant energy waste and, consequently, a decrease in ablation efficiency rather than an increase. To more accurately quantify the material removal effect under different powers, the material removal rate is introduced as an evaluation metric. The calculation formula is shown in Equation (1).

(1) $$\mathrm{MRR}=A_{\mathrm{cross}}\cdot \nu$$

where $A_{cross}$ represents the cross-sectional area and v is the laser scanning speed. Using the average cross-sectional profiles and Origin software 2021, the area below (under the baseline) in the cross-sectional profiles is quantitatively calculated. The results show that the cross-sectional area reaches a maximum of 183.72 ± 9.19 μm^2^ at 9 W; the cross-sectional areas at 3 W, 15 W, and 21 W are 85.16 ± 4.26 μm^2^, 158.79 ± 8.21 μm^2^, and 165.40 ± 8.47 μm^2^, respectively. Based on Equation (1), the material removal rates at the different powers are calculated to be 0.043 mm^3^/s, 0.092 mm^3^/s, 0.079 mm^3^/s, and 0.083 mm^3^/s. Statistical analysis (*t*-test) reveals that the material removal rate at 9 W is significantly greater than those at 3 W (t = 23.45, *p* < 0.05), 15 W (t = 6.61, *p* < 0.05), and 21 W (t = 4.13, *p* < 0.05), respectively. These results indicate that the material removal rate is highest at 9 W, while it slightly decreases at higher powers. This trend is consistent with the variation in ablation depth, further confirming the negative impact of bubble aggregation on processing efficiency at elevated laser powers.

**Figure 7:**
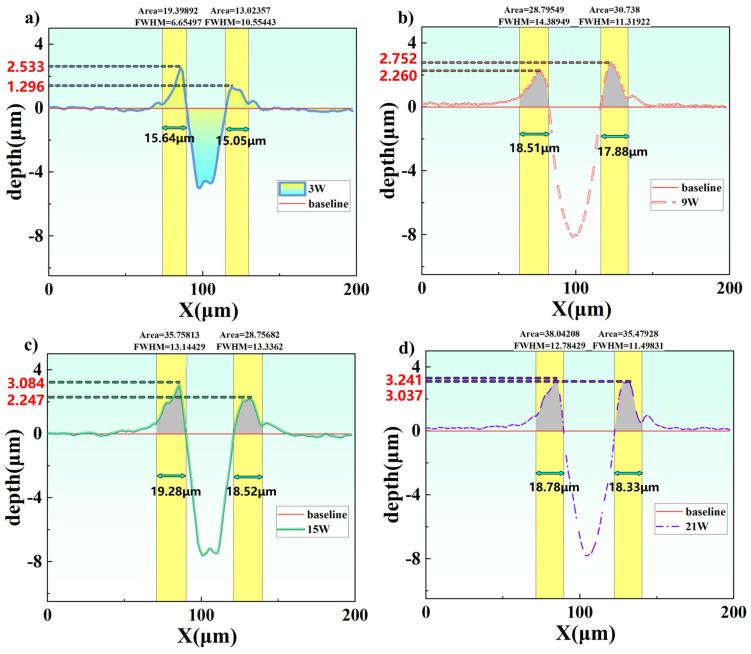
Ablation cross-sections at 0° for different power levels: (**a**) 3 W, (**b**) 9 W, (**c**) 15 W, (**d**) 21 W.

To analyze the processing differences under different laser powers in more detail, we start from the cross-sectional profiles and further examine the variation characteristics of the recast layer; the results are shown in Figure 7.

It is observed that as the laser power increases, the height of the recast layer continuously rises: from a maximum of 2.53 μm at 3 W to 2.75 μm, 3.08 μm, and 3.24 μm at 9 W, 15 W, and 21 W, respectively. The thickness of the recast layer increases from 15.64 μm at 3 W to 19.28 μm at 15 W during the initial power increase, and then remains stable at approximately 19 μm when the power is further increased to 21 W. Meanwhile, the cross-sectional area of the recast layer continuously increases from 19.40 μm^2^ at 3 W to a maximum of 38.04 μm^2^ at 21 W. Under all power conditions, the recast layer exhibits a symmetric distribution on both sides of the machined groove.

The above phenomena are attributed to the following factors. First, higher power implies greater energy input per unit time, leading to intensified material melting and vaporization; the ejected molten material rapidly cools and solidifies in the underwater environment to form a recast layer. Water has a significantly higher thermal conductivity than air, allowing it to quickly absorb heat from the molten material and suppress excessive accumulation. Even at low powers where a stable jet does not yet form, the shock waves generated by the laser itself induce fluid motion, carrying away some debris and further limiting the thickness of the recast layer. Second, under normal incidence (0°), the laser energy follows a Gaussian symmetric distribution—rotationally symmetric about the optical axis, with maximum energy at the center and uniform attenuation toward the periphery. This ensures identical heating and melting conditions on both sides of the groove, resulting in a symmetric recast layer.

Third, the recast layer mainly builds up upward rather than spreading significantly sideways. This is determined by the combined effects of rapid underwater solidification, surface tension, directional action of shock waves, and the Gaussian energy distribution. The high thermal conductivity of water causes the ejected molten material to solidify quickly, leaving no time for lateral spreading. Meanwhile, the surface tension of the melt tends to form a convex structure with small curvature to reduce surface energy, making it easier to stack vertically on top of the already solidified layer. Under high power, the laser-induced shock waves and cavitation bubble collapse produce micro-jets directed toward the workpiece surface, squeezing the melt toward the center of the groove rather than pushing it sideways. In addition, under normal incidence, the Gaussian beam has the highest energy at the center, so the melt is ejected upward from the bottom; the sidewalls have a large temperature gradient and solidify extremely quickly, preventing lateral flow. Subsequent scanning preferentially heats the already existing protruding areas, further enhancing vertical accumulation. Therefore, the height and cross-sectional area of the recast layer continuously increase, while the lateral thickness quickly stabilizes, exhibiting a symmetric buildup pattern dominated by upward growth.

### 3.2. Influence of Incidence Angle on Jet Behavior and Macroscopic Surface Morphology

#### 3.2.1. Effect of Incidence Angle on Jet Morphology

**Figure 8:**
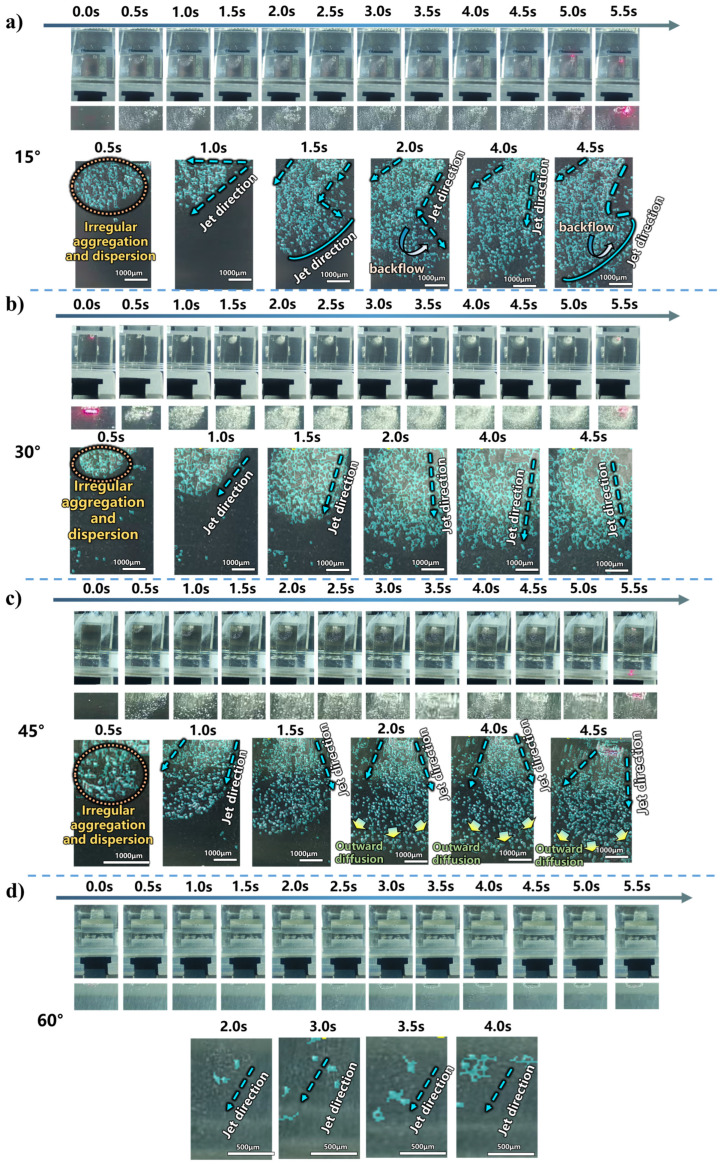
Jet evolution and jet velocity at different incidence angles (15 W, 1 mm liquid thickness): (**a**) 15°, (**b**) 30°, (**c**) 45°, (**d**) 60°.

As the laser power increases, the average maximum ablation depth of the machining groove and the material removal rate show a decreasing trend, while the surface morphology quality deteriorates due to debris spattering and oxidation. As analyzed in the previous section, under normal incidence (0°), this is attributed to disordered bubble generation, accumulation of secondary bubbles on the surface, and significant randomness in the jet direction. To improve the processing performance, this section investigates the regulation of cavitation bubble dynamics by the solid boundary through adjusting the sample inclination angle, thereby optimizing the laser-induced microjet behavior. Based on the optimized parameters from the previous section where a stable jet is formed, the experiments are conducted with a fixed laser power of 15 W and a liquid layer thickness of 1 mm. Using an adjustable angle gauge, the sample inclination angles are set to 15°, 30°, 45°, and 60°. Employing the OpenCV image processing tool described in the previous section, frames are extracted from the recorded processing videos at 0.5 s intervals. Specific time nodes are selected for processing to analyze the dynamic evolution of the laser-induced microjet at different angles. The evolution of jet morphology at each angle is shown in Figure 8.

First, it is observed that at different angles, the jet generation stage consistently begins with disordered bubble disturbances, gradually evolving into an ordered, directional jet morphology as processing proceeds. This occurs because, in the initial stage of laser action, no stable cavitation nuclei exist in the liquid medium. The laser energy first ionizes the liquid in the focal region through nonlinear absorption, forming randomly distributed initial cavitation nuclei. The size, position, and generation timing of these nuclei exhibit significant randomness, leading to chaotic bubble generation and collapse in the initial stage, manifested as disordered jet morphology and dispersed directionality. As laser pulses continue, a stable cavitation bubble generation region gradually forms in the liquid. Bubbles are continuously attracted toward the solid boundary, while the repulsive effect of the free boundary limits their vertical movement space, preventing bubbles from floating upward due to buoyancy. This causes the energy release direction during bubble collapse to gradually converge toward the wall normal direction. When the distance between bubbles and the two boundaries reaches dynamic equilibrium, the vector synthesis of Bjerknes forces stabilizes, and the jet direction becomes locked near the wall normal, forming a stable directional jet mode. The jet direction thus completes its convergence, as shown in the OpenCV processed images at 0.5–1.0 s in Figure 8a–c.

**Figure 9:**
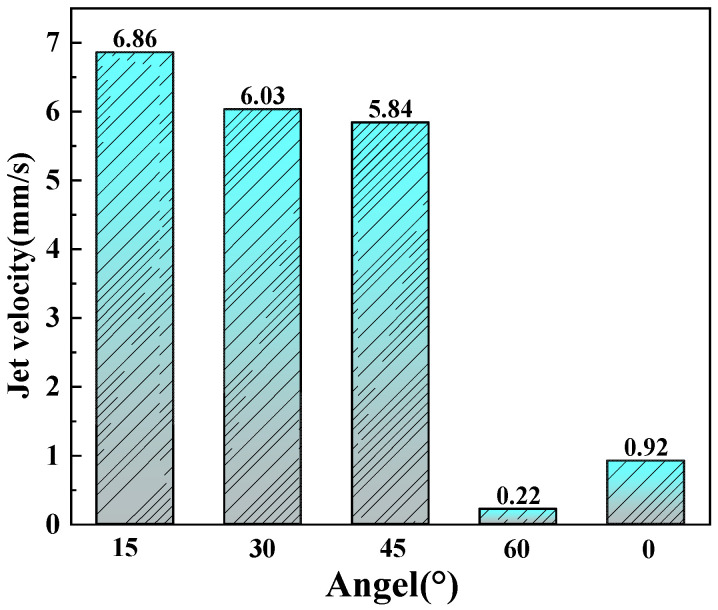
Diagram of jet velocity at different angles under 15 W and 1 mm.

At a 60° inclination angle, no significant jet is generated; only sporadic individual cavitation bubbles are observed (Figure 8d). This is because at 60°, the laser spot projection on the workpiece surface is significantly elongated, substantially increasing the spot area while the total laser power remains constant, resulting in a marked decrease in energy density per unit area. This reduction in energy density directly weakens the laser’s ability to ionize the liquid and generate cavitation bubbles, making bubble formation difficult and unstable, occurring only sporadically in regions with local energy fluctuations. Furthermore, insufficient energy input means that even if a few bubbles are generated, their collapse process lacks sufficient energy to form an impactful directional jet. Jet velocity measurements show that at 60°, the jet velocity is only 0.22 mm/s (Figure 9), further confirming jet failure at this angle.

Within the 15–45° range, jet convergence and straightness gradually decrease, as shown in the OpenCV processed images at 1.5–2.0 s in Figure 8a–c. This is primarily because, as the angle increases, the reduced energy input directly affects cavitation bubble collapse intensity—the potential energy stored in bubbles decreases, and the energy released during collapse correspondingly weakens, leading to reduced initial jet velocity and deteriorated directionality. Quantitative analysis of jet velocity shows values of 6.86 mm/s, 6.03 mm/s, and 5.84 mm/s at 15°, 30°, and 45°, respectively (Figure 9), exhibiting a decreasing trend. Simultaneously, as the spot area elongates with increasing angle, energy becomes spatially dispersed, further reducing local energy density and making it difficult for bubbles to collapse stably at fixed positions. The attenuation at the energy level combines with changes in Bjerknes force direction: at small angles, the normal force dominates, ensuring good jet directionality; as the angle increases, the tangential component increases, causing jet direction deflection, manifested as decreased convergence and straightness.

It is also observed that at 15°, the jet at the processing end exhibits a “mushroom cap”-like backflow (Figure 8a, 4.0–4.5 s). However, when the angle increases to 30–45°, the jet at the processing end becomes divergent, with slight bubble aggregation at 30° and secondary bubbles floating around (Figure 8b,c, 4.0–4.5 s). This is because at small angles, the high-velocity jet forms natural vortices after overcoming liquid resistance—the jet velocity of 6.86 mm/s at 15° indicates sufficient energy to drive fluid in an organized backflow after completing the primary impact. As the angle increases, the reduced initial jet velocity (6.03 mm/s at 30° and 5.84 mm/s at 45°) results in insufficient momentum during downward propagation, preventing the formation of organized “mushroom cap” backflow and leading only to chaotic divergent dispersion.

#### 3.2.2. Effect of Incidence Angle on Ablation Morphology and Resultant Surface Characteristics

**Figure 10:**
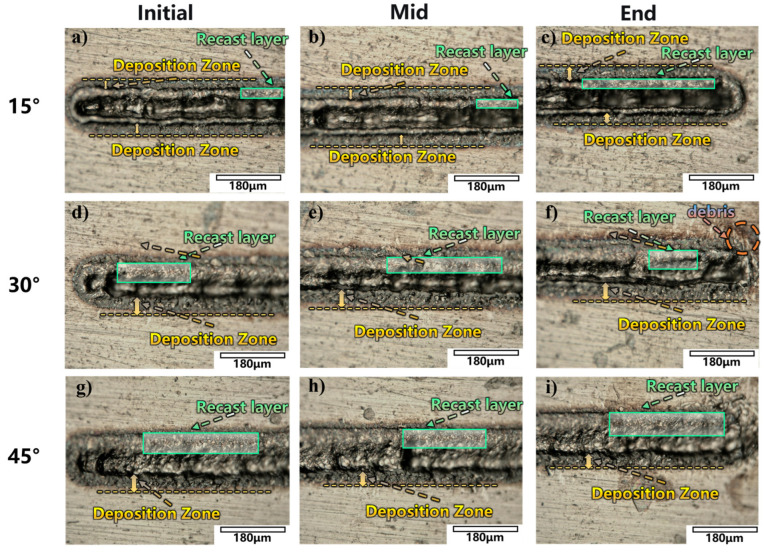
Macroscopic morphologies of the machined surface at different angles at 1 mm and 15 W: (**a**–**c**) 15°, (**d**–**f**) 30°, (**g**–**i**) 45°.

In the previous section, the morphological evolution of the jet at different angles under selected power parameters is discussed and analyzed, including changes in jet straightness, convergence, and end morphology, and the jet velocity is quantitatively calculated. The results indicate that at a 15° incidence angle, the jet exhibits better straightness and convergence. This section focuses on the macroscopic effects of different angles on multi-position (head, middle, tail) laser ablation under the conditions of a 1 mm liquid layer thickness and 15 W laser power, and the results are shown in Figure 10.

It is clearly observed that when the sample is tilted at a certain angle, a directional jet forms. At this point, the deposition area of ablation debris at the machining edge significantly decreases. Simultaneously, due to the directional ejection of the jet, the debris deposition notably transforms from the splashing pattern observed at 0° into a distinct deposition band along the laser ablation direction. This phenomenon is particularly evident in the middle region images (Figure 10b,e,h). This occurs because, at inclined angles, the jet energy is highly concentrated in a single direction, continuously scouring along the wall surface and directionally transporting ablation debris downstream. Consequently, the jet no longer dissipates energy in all directions, and debris is effectively transported in a concentrated manner rather than being randomly deposited, leading to a significant reduction in the deposition area at the processing edge. The debris gradually settles along a fixed path, ultimately converging into a clear deposition band aligned with the laser ablation direction.

Furthermore, Figure 10 reveals significant morphological differences at the processing end for different angles. At 30° and 45°, distinct ablation debris and splashing deposition bands appear at the processing end (Figure 10f,i), while at 15°, the processing end exhibits a clean surface morphology (Figure 10c). This phenomenon is attributed to the “end effect” in laser processing, combined with the regulation of energy input and cavitation behavior by the angle.

During laser scanning processing, the ablation morphology near the start and end points of the scanning path often differs from the stable intermediate region—this is known as the end effect. Its core lies in heat accumulation: as scanning proceeds, heat continuously accumulates around the laser spot. When the spot reaches the end point and stops, the heat loses its forward diffusion path, resulting in exceptionally concentrated energy deposition at the final spot position. Moreover, the surrounding material has been preheated by preceding pulses, leading to sharply increased material removal at the end point and forming a deeper terminal pit.

In this experiment, the end effect causes significantly elevated heat accumulation at the processing tail, directly affecting cavitation bubble generation and motion. According to cavitation theory, increased temperature lowers the energy threshold required for cavitation bubble generation in liquids [23]. Combined with the previously described end effect, this results in more cavitation bubbles being generated at the tail compared to other processing regions. However, as the incidence angle increases to 30° and 45°, the laser energy density attenuates, leading to decreased jet velocity and reduced debris removal capacity. Some bubbles and ablation debris at the tail cannot be effectively carried away by the jet and remain at the processing end. These retained bubbles collapse in situ under subsequent pulses, and the released jets carry ablation debris, ultimately forming the observed splashing deposition bands. Additionally, these bubbles that accumulate and collapse chaotically at the end scatter and shield the incident laser, disrupting stable laser transmission and causing the actual ablation path to deviate from the intended straight line, further deteriorating processing quality at the end region.

In contrast, at 15°, sufficient energy and stable jet formation allow timely removal of excess bubbles generated by heat accumulation. The end effect is effectively suppressed, maintaining a clean morphology at the processing end.

**Figure 11:**
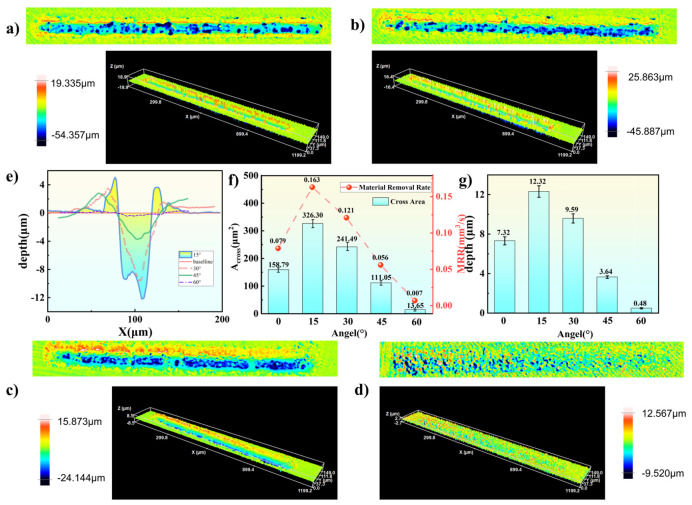
White light interferometry morphologies of ablation results under different incidence angles: (**a**) 3 W, (**b**) 9 W, (**c**) 15 W, (**d**) 21 W, (**e**) average cross-sectional ablation profiles, (**f**) material removal rate and Groove cross-sectional area and (**g**) average maximum ablation depth.

Building on these observations, the three-dimensional morphologies of the machined grooves are further analyzed to quantitatively assess the effects of different inclination angles. The three-dimensional machining morphologies at different angles are shown in Figure 11a–d.

At an inclination angle of 15°, the recast layer is thinnest and exhibits the smallest distribution range on both the upper and lower edges. Overall observation of the four sets of machining results reveals that the recast layer thickness increases progressively toward the scanning end, which corroborates the previous analysis.

As the inclination angle increases, the maximum single-point ablation depth decreases significantly: from 54.357 μm at 15° to 9.520 μm at 60°. Correspondingly, the continuity and uniformity of the ablated lines also change. At a small angle of 15°, the machined trajectory is a continuous, uniform, and clearly defined straight line, indicating optimal material removal. When the angle increases to 45°, machining uniformity decreases, and intermittent discontinuities appear in the middle of the ablated region. At 60°, the effective energy density under oblique incidence decreases drastically, becoming insufficient to induce stable material vaporization. Consequently, no continuous, distinct machined groove forms on the workpiece surface. Due to the “end effect,” only minimal material removal occurs at the end of the scanning trajectory, indicating that effective linear material removal is unachievable at this angle.

Based on the average cross-sectional profiles (Figure 11e), the machined cross-section at 15° exhibits a “straight-walled” shape without noticeable inclination. At 30° and 45°, the cross-sections transition to a “V-shape,” with progressively decreasing aspect ratios and a rightward shift in the machined end as the angle increases. This reflects the elongation of the laser spot in the y-direction and the expansion of the irradiated area. The average maximum ablation depth peaks at 15° with a value of 12.32 ± 0.58 μm, followed by 9.59 ± 0.46 μm at 30°, 3.64 ± 0.13 μm at 45°, and 0.48 ± 0.06 μm at 60°. Compared to the value of 7.32 ± 0.41 μm at 0°, the average maximum depths at 15° and 30° increase by 68.3% and 31.0%, respectively. Statistical analysis (*t*-test) further confirms that the ablation depth at 15° is significantly greater than those at 0° (t = 6.39, *p* < 0.05), 30° (t = 6.36, *p* < 0.05), 45° (t = 21.16, *p* < 0.05), and 60° (t = 33.98, *p* < 0.05).

Using the processing method described previously and the material removal rate calculation formula (Equation (1)), the cross-sectional areas and material removal rates at different angles are calculated. The results show that the cross-sectional area is largest at 15°, reaching 326 ± 16.10 μm^2^; at 30°, 45°, 60°, and 0°, the cross-sectional areas are 241.49 ± 14.76 μm^2^, 111.05 ± 5.75 μm^2^, 13.65 ± 1.26 μm^2^, and 158.79 ± 3.76 μm^2^, respectively. Statistical analysis (*t*-test) indicates that the cross-sectional area at 15° is significantly greater than those at 30° (t = 6.73, *p* < 0.05), 45° (t = 21.82, *p* < 0.05), 60° (t = 33.55, *p* < 0.05), and 0° (t = 17.54, *p* < 0.05). Based on Equation (1), the material removal rates at each angle are calculated to be 0.163 mm^3^/s (15°), 0.121 mm^3^/s (30°), 0.056 mm^3^/s (45°), and 0.007 mm^3^/s (60°). Compared to the material removal rate of 0.079 mm^3^/s at 0°, the rates at 15° and 30° increase by 106.3% and 53.1%, respectively. This further demonstrates that at an inclination angle of 15°, the generated directional microjet can more effectively assist the machining process, thereby significantly enhancing ablation efficiency.

**Figure 12:**
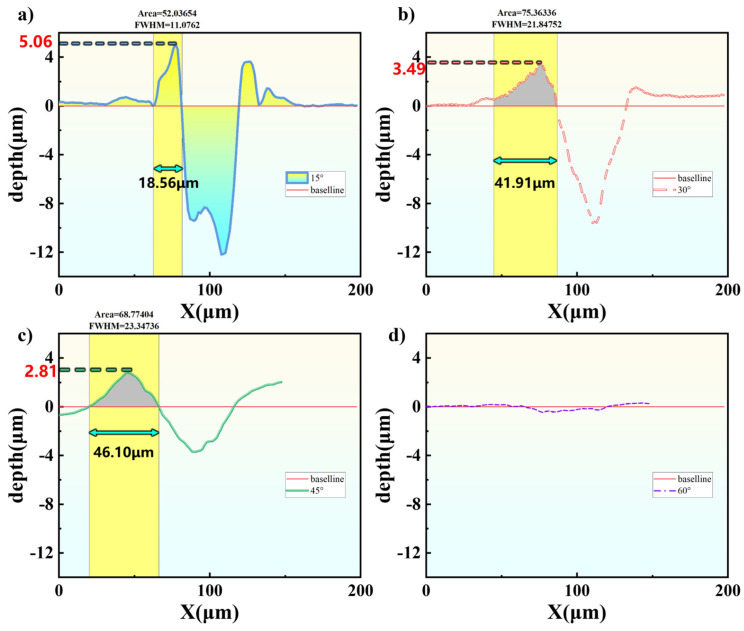
Ablation cross-sections at 15 W for different angles: (**a**) 15°, (**b**) 30°, (**c**) 45°, (**d**) 60°.

To analyze the processing differences under different inclination angles in more detail, we start from the cross-sectional profiles and further examine the variation characteristics of the recast layer; the results are shown in Figure 12. It is observed that as the inclination angle increases, the height of the recast layer continuously decreases: from a maximum of 5.06 μm at 15° to 3.49 μm at 30° and 2.81 μm at 45°, while at 60° no obvious recast layer is formed. The thickness of the recast layer increases significantly with increasing angle: from 18.56 μm at 15° to 41.91 μm at 30° and further to 46.10 μm at 45°. Overall, the recast layer exhibits a “low and wide” trend. The cross-sectional area of the recast layer first increases and then slightly decreases: from 52.04 μm^2^ at 15° to 75.36 μm^2^ at 30°, and then drops slightly to 68.77 μm^2^ at 45°.

It is worth noting that the recast layer is not symmetrically distributed on both sides of the machined groove. Post-processing observations reveal that the recast layer thickness on the upper edge is significantly greater than that on the lower edge. At 15°, the recast layer on the upper edge is thinnest, and its thickness progressively increases with larger inclination angles. This phenomenon is explained from two aspects. First, the energy distribution of obliquely incident laser exhibits asymmetry—along the incident direction (lower edge), the energy gradient is gentler and the effective range extends farther, allowing material to be fully vaporized and ejected. In contrast, opposite the incident direction (upper edge), the energy gradient is steeper with rapid attenuation, resulting in incomplete material removal and residual molten material that solidifies. Second, the directional jet scours downward along the inclined direction, transporting most debris to the lower edge region, resulting in a thinner recast layer there. The upper edge, being in the “upstream” region of the jet scouring, is not only difficult to clean effectively but also receives some debris deposition due to backflow vortices. Consequently, as the inclination angle increases, the recast layer thickness on the upper edge continuously increases, while the lower edge remains thin. At 60°, because the jet direction is almost parallel to the surface, no obvious continuous recast layer is formed.

### 3.3. Influence of Incidence Angle on Microstructure and Elemental Composition

**Figure 13:**
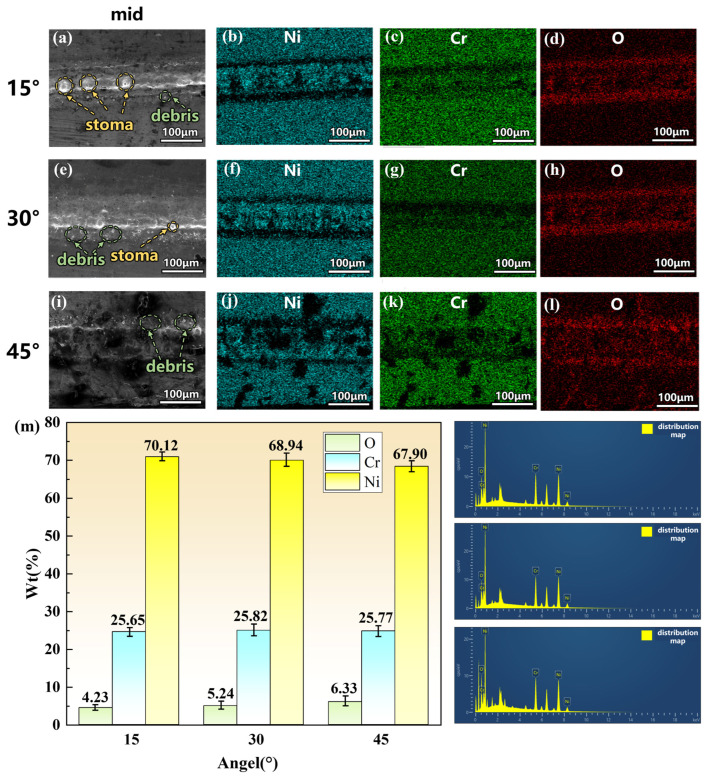
SEM images and elemental composition analysis of the middle region at different angles: (**a**–**d**) 15°, (**e**–**h**) 30°, (**i**–**l**) 45°, (**m**) elemental composition content.

In the previous section, the ablation morphology at different angles is analyzed from a macroscopic perspective, with quantitative comparisons of the average ablation depth and the material removal rate relative to that at 0°. In this section, scanning electron microscopy (SEM) is employed to observe the microscopic morphology of the middle region of the processed grooves at different angles, and energy-dispersive X-ray spectroscopy (EDS) is used to quantitatively analyze the differences in Ni, Cr, and O content in the middle region. The results are shown in Figure 13**.**

SEM observation of the middle region of the specimens reveals differences in ablation morphology under different laser incidence angles, as shown in Figure 13a,e. Holes are observed at the bottom of the ablated regions at 15° and 30°, which can be attributed to the occurrence of “phase explosion” during underwater processing [24]. When the laser irradiates the surface, the material absorbs extremely high energy, causing the temperature to rapidly rise above the boiling point and forming a non-equilibrium superheated liquid. As the temperature approaches the thermodynamic critical point, homogeneous nucleation occurs within the liquid, with numerous tiny vapor nuclei forming and coalescing within an extremely short time. This leads to a sharp increase in local pressure, ultimately triggering explosive boiling that ejects molten material in the form of high-velocity micro-jets, thereby leaving holes at the bottom of the ablation zone.

Further EDS analysis reveals that at 15° and 30°, oxygen (O) is mainly enriched at both sides of the ablated grooves. At 45°, however, some oxygen also appears in the non-machined areas (Figure 13d,h,l). This is because, as the incidence angle increases, the velocity of the directional microjet decreases, reducing its capacity to carry ablation products. Consequently, partially oxidized ablation debris cannot be effectively expelled from the processing zone and instead deposits on the surrounding non-machined surface. Quantitative comparison of oxygen content at the three angles shows the lowest value at 15° (4.35% ± 0.15%), with values increasing to 5.23% ± 0.18% at 30° and 6.33% ± 0.22% at 45°. Statistical analysis (*t*-test) reveals that the oxygen content at 15° is significantly lower than that at 30° (t = 5.96, *p* < 0.05) and at 45° (t = 13.00, *p* < 0.05). The oxygen content can, to some extent, characterize the thickness of the recast layer and the degree of residual oxidation products. Its sources include two aspects: the release of dissolved oxygen during the collapse of laser-induced cavitation bubbles, and the ionization of water molecules by high-energy laser irradiation, generating oxygen radicals that participate in oxidation reactions in the ablation zone.

(2) $$4\mathrm{Cr}+3O_{2}\to {2\mathrm{Cr}}_{2}O_{3}$$

The reduction in Cr content indirectly confirms that more material is removed from the target region at 15°. Meanwhile, the lowest O content at this angle further demonstrates the enhanced capability of the jet to carry away ablation debris, resulting in a thinner recast layer compared to other angles.

## 4. Discussion

**Figure 14:**
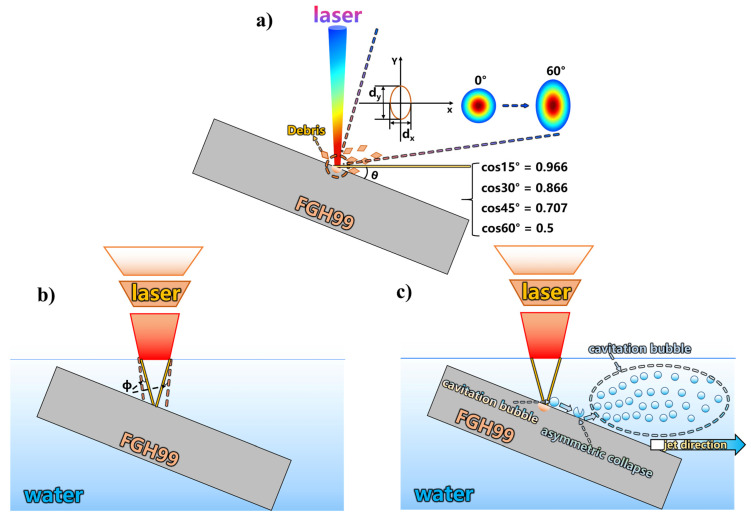
Schematic diagram of the material removal mechanism at different angles: (**a**) spot variation, (**b**) liquid layer effect, (**c**) induced jet formation.

To better analyze the material removal mechanism of laser ablation at different angles, the discussion is carried out from the following three aspects: analysis of multi-angle laser ablation in air, the weakening effect of the liquid layer on the laser, and bubble evolution and directional jet generation, as shown in Figure 14.

When the sample surface forms a certain angle with the laser optical axis, the projection pattern of the laser beam on the material surface undergoes significant changes, which is the primary consideration for understanding the mechanism of oblique incidence laser processing. Under normal incidence conditions, a circular Gaussian spot forms a symmetrical energy distribution on the workpiece surface. However, when the laser is incident at an angle (i.e., the angle between the laser optical axis and the normal to the material surface), the spot shape undergoes geometric distortion, gradually evolving from a circle to an ellipse. Specifically, in the direction perpendicular to the plane of incidence (defined as the X-direction), the spot size remains constant; while in the direction parallel to the plane of incidence (defined as the Y-direction), the spot size is elongated as the incidence angle increases. This geometric relationship can be precisely described by the formula Equation (3) [25].

(3) $$\left\{\begin{array}{ll} d_{x}=2\omega_{0} & \\ d_{y}=\frac{2\omega_{0}}{\cos\theta } & \end{array}\right.$$

where ω_0_ is beam waist radius of the laser, $d_{x}$ and $d_{y}$ represent the minor and major axis lengths of the elliptical spot, respectively. According to Equation (3), as the incidence angle θ increases, the major axis $d_{y}$ is monotonically elongated, while the minor axis $d_{x}$ remains constant. This implies that when the incidence angle increases from 0° to 60°, the spot length in the direction doubles, resulting in a significant increase in the overall spot area.

In laser processing, the spatial distribution of energy within the spot is a critical factor determining the ablation morphology. For a fundamental Gaussian beam, the energy density distribution on the material surface under normal incidence is described by the standard Gaussian function, as shown in Equation (4):

(4) $$F(z,r)=F_{0}\exp\left(\frac{-2r^{2}}{\omega {\left(z\right)}^{2}}\right)$$

where $F_{0}$ is the peak energy density and *r* is the distance from the point of interest to the optical axis. The peak energy density $F_{0}$ is related to the laser power P, repetition frequency f, and beam waist radius $\omega \left(z\right)$ by Equations (5) and (6):

(5) $$F_{0}=\frac{2P}{f\pi \omega {\left(z\right)}^{2}}$$

(6) $$\omega (z)=\omega_{0}\sqrt{1+{\left(\frac{\lambda z}{\pi \omega_{0}^{2}}\right)}^{2}}$$

Substituting Equations (5) and (6) into Equation (2) and converting to Cartesian coordinates, the energy density distribution for normal incidence can be further expressed as Equation (7):

(7) $$F(x,y,z)=\frac{2P}{f\pi \omega {\left(z\right)}^{2}}\exp\left(\frac{-2(x^{2}+y^{2})}{\omega {\left(z\right)}^{2}}\right)$$

Equation (7) clearly reveals two essential characteristics of a normally incident Gaussian beam: first, the peak energy density $F_{0}$ determines the energy intensity at the spot center; second, the Gaussian function in the exponential term describes the energy decay from the center outward, and due to the symmetry in  x  and  y, the energy distribution exhibits isotropic circular symmetry.

When the laser is incident obliquely, the description of the energy distribution becomes more complex. By establishing the transformation relationship between the optical axis coordinate system and the workpiece coordinate system, introducing spot radius functions in two directions [26,27], the energy density distribution function for an obliquely incident Gaussian beam on the workpiece surface is derived. After transforming the coordinate system to the workpiece surface and simplifying the focal position (assuming the laser focus is on the workpiece surface), the energy distribution under oblique incidence is expressed as Equation (8) [28]:

(8) $$F_{0}(x,y,z)=\frac{2P\cos\theta }{f\pi \omega_{0}^{2}\sqrt{1+{\left(\frac{\lambda y_{0}\sin\theta }{\pi \omega_{0}^{2}}\right)}^{2}}}\exp\left(-2\left(\frac{x_{0}^{2}}{\omega_{0}^{2}}+\frac{y_{0}^{2}\cos^{2}\theta }{\omega_{0}^{2}\left(1+{\left(\frac{\lambda y_{0}\sin\theta }{\pi \omega_{0}^{2}}\right)}^{2}\right)}\right)\right)$$

Comparing Equation (7) for normal incidence with Equation (8) for oblique incidence reveals their essential differences. At the spot center (x = 0, y = 0), Equation (9) simplifies to:

(9) $$F_{peak}(\theta )=\frac{2Pcos\theta }{f\pi {\omega_{0}}^{2}}=F_{0}\cos(\theta )$$

**Figure 15:**
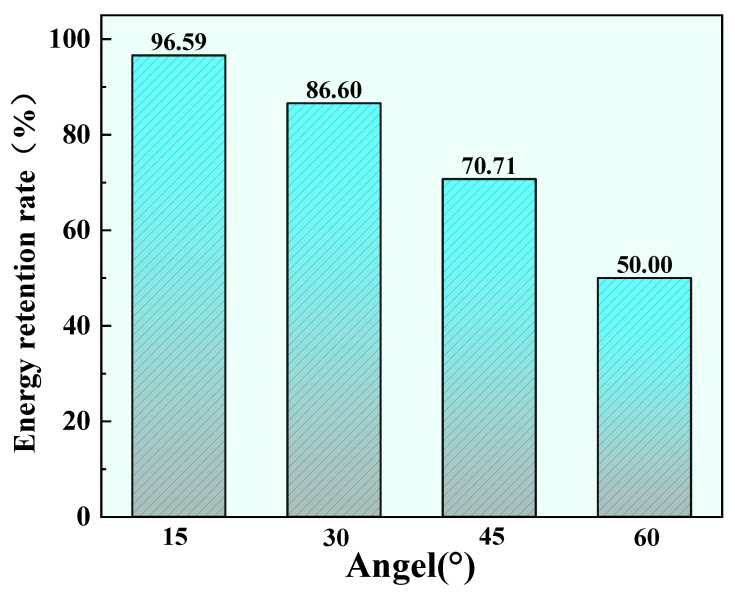
Energy retention rate at different angles.

This indicates that under oblique incidence, the peak energy density at the center decreases according to the cosine of the incidence angle. This concise quantitative relationship implies that at 15° incidence, the peak energy retains 96.59% (a mere 3.31% decrease); at 30°, it retains 86.60% (a 13.4% decrease); at 45°, it retains 70.71% (a 29.29% decrease); and when the incidence angle increases to 60°, the peak energy is only 50.00% of the normal incidence value, an attenuation of half, as shown in Figure 15. This energy attenuation law provides the energetic basis for understanding the decline in processing efficiency at large angles.

More critically, Equation (8) reveals the spatial asymmetry of the energy distribution for obliquely incident lasers. From the perspective of directional energy distribution, the obliquely incident laser exhibits distinctly different characteristics along the minor axis (X-direction) and major axis (Y-direction). Along the minor axis, the energy distribution retains the basic Gaussian form, but the peak intensity is reduced by a factor of cosθ. Along the major axis, however, the energy distribution is not only geometrically stretched but also exhibits significant elongation and asymmetry due to the presence of the radical term $\sqrt{1+{\left(\frac{\lambda y_{0}\sin\theta }{\pi w}\right)}^{2}}$. This radical term causes the energy decay rate in the Y-direction to vary with the Y-coordinate rather than remaining constant, leading to a deviation from the ideal Gaussian form and an overall flattening of the distribution. This flattened energy distribution results in an elongated elliptical ablation morphology, contrasting sharply with the circular Gaussian distribution under normal incidence. The energy exhibits stretching and asymmetric deformation in the plane of incidence, a characteristic that directly determines the geometry of the ablation crater and sets the initial conditions for subsequent physical processes.

When the processing environment transitions from air to a liquid layer, the liquid layer refracts and attenuates the laser. Taking water as an example, the refraction angle of the laser can be calculated using Snell’s law. As shown in Equation (10):

(10) $$\Phi =sin^{\left(-1\right)}\left\{\frac{sin\left[\tan^{-1}(D/2F)\right]n_{a}}{n_{w}}\right\}$$

where D is the diameter of the collimated laser beam entering the focusing lens, F is the focal length of the focusing lens, $n_{a}$ is the refractive index of air (1.0), and $n_{w}$ is the refractive index of water (1.333) [29].

According to the Beer-Lambert law [30], the attenuation of light intensity propagating through a medium can be expressed as Equation (11):

(11) $$I(z)=I_{0}\cdot \exp(-\alpha z)$$

where $I_{0}\;$ is the incident light intensity, $I\left(z\right)$ is the intensity after propagating a distance z, and a is the linear absorption coefficient of the medium. The absorption coefficient of water is x = 0.135 cm^−1^ [31]. The transmittance T  of the water layer is given by Equation (12):

(12) $$T=\frac{I(z)}{I_{0}}=\exp\left(-\alpha \cdot h\right)$$

Calculation shows that under normal incidence, the transmittance of the water layer is 98.66%. When the sample is tilted, the actual propagation distance of the laser within the water layer becomes h/cosθ. Therefore, a correction factor must be introduced, and the transmittance expression is modified to Equation (13):

(13) $$T=\frac{I(z)}{I_{0}}=\exp\left(-\alpha \cdot \frac{h}{\cos\theta }\right)$$

Calculating the transmittance for incidence angles of 15°, 30°, 45°, and 60° yields 98.61%, 98.45%, 98.11%, and 97.34%, respectively. These results indicate that the attenuation effect of the water layer on the laser varies only slightly with the sample inclination angle and does not constitute a dominant factor influencing the processing outcome.

When the nanosecond laser passes through the water layer and irradiates the surface of the FGH99 superalloy, the laser energy is absorbed by the material, causing instantaneous local temperature rise, melting, and even vaporization, forming molten material and ablation debris. Concurrently, the laser breaks down the liquid medium, generating cavitation bubbles. Near the solid boundary, these cavitation bubbles undergo asymmetric collapse. This collapse process not only induces high-speed microjets but is also accompanied by the generation and collapse of secondary bubbles. This complex fluid dynamic behavior is described using the Kelvin impulse principle, as shown in Equation (14) [32]:

(14) $$I=\int_{0}^{t}F(t)\mathrm{dt}$$

where $F\left(t\right)$ is the force acting on the cavitation bubble in the liquid, as shown in Equation (15):

(15) $$F(t)=\rho gVe-\frac{\rho }{16\pi h_{r}^{2}}{\left(\frac{dV}{dt}\right)}^{2}$$

in this equation, ρ is the fluid density, V is the bubble volume, e is the unit vector in the buoyancy direction, $h_{r}$ is the distance from the cavitation bubble to the boundary. The first term in Equation (12) represents the buoyancy force acting on the bubble, while the second term is the Bjerknes force—a force arising from the dynamic attraction of a pulsating bubble by the solid boundary. Its direction is always perpendicular to the wall and inward, drawing the bubble toward the wall during the collapse phase. The vector competition between buoyancy and the Bjerknes force collectively determines the bubble’s trajectory, collapse location, and energy release direction, thereby directly influencing the formation angle and velocity magnitude of the resulting microjet.

When the high-speed microjet generated by the collapsing cavitation bubble impacts the ablation region, its primary role is not to directly deepen the ablation crater. Instead, it acts like a “water jet” vigorously scouring and removing molten material and debris from the crater bottom and walls, transporting them away from the processing zone. This process can be described by the water hammer formula Equation (16):

(16) $$P_{hammer}=\rho cv_{j}$$

where ρ is the liquid density, c is the speed of sound in the liquid, and $\nu_{j}$ is the jet velocity. Combined with the previous analysis of jet behavior at different angles, it is known that the jet velocity reaches its peak at a 15° incidence angle. This implies that the water hammer pressure is maximized at this angle, providing the strongest impact and displacement capability for removing molten material. Furthermore, at 15°, the jet exhibits the highest straightness, the smallest divergence angle, and no backflow, ensuring concentrated energy and stable directionality, leading to efficient and orderly debris removal. This timely debris clearance eliminates the shielding layer between the laser and the material, allowing subsequent laser pulses to consistently act on the target area and preventing energy absorption and scattering by debris. Consequently, this results in a significant enhancement of both ablation depth and material removal rate. Therefore, 15° is identified as the optimal angle for laser-induced microjet-assisted ablation of FGH99. The underlying mechanism lies in the synergistic optimization of jet velocity, directionality, and stability, which is a coupled outcome of efficient energy input and effective debris removal.

## 5. Conclusions

(1) Under a liquid layer thickness of 1 mm and a laser power of 15 W, a vertically symmetric conical jet morphology was formed. Under normal incidence, the maximum material removal rate of 0.092 mm^3^/s was achieved at 9 W. However, as the laser power further increased, the material removal rate did not improve correspondingly but instead decreased, indicating that bubble aggregation at higher powers negatively affected processing efficiency.
(2) At a sample inclination angle of 15°, the jet exhibited the maximum velocity, along with the best straightness and convergence, reaching a jet velocity of 6.86 mm/s. At this angle, the jet demonstrated optimal stability and directionality, providing favorable dynamic conditions for efficient debris removal.
(3) Under the processing conditions of 1 mm liquid thickness and 15 W laser power, the sample inclination angle significantly influenced the material removal rate. Compared to 0.079 mm^3^/s at 0°, the material removal rates at 15° and 30° increased by 106.3% and 53.1%, reaching 0.163 mm^3^/s and 0.121 mm^3^/s, respectively. This demonstrated that the directional microjet generated by optimizing the inclination angle could effectively assist the ablation process, significantly enhancing material removal efficiency.
(4) EDS analysis revealed the lowest oxygen content (4.35 ± 0.15%), indicating the strongest debris removal capability and the thinnest recast layer. Conversely, higher oxygen content at 30° (5.23 ± 0.18%) and 45° (6.33 ± 0.22%) suggested less effective debris removal and greater surface oxidation. These microscopic results validated 15° as the optimal angle for laser-induced microjet-assisted ablation of FGH99.

As a novel laser processing technology, laser-induced microjet-assisted ablation was systematically investigated in this study, revealing the influence mechanisms of different laser incidence angles on material removal and achieving the optimization of process parameters. The findings provided important theoretical foundations and practical guidance for surface microstructuring (anti-icing performance enhancement) and efficient material removal (film cooling hole drilling) of high-temperature alloys in the aerospace industry.

## Figures and Tables

**Table 1:** Element composition percentage of FGH99 alloy.

C	Cr	B	Fe	Mo	Al	Ti	Nb	W	Ni
0.03	20	13	4.3	2.9	3.6	3.5	1.5	0.35	Other

**Table 2:** The parameters for laser ablation.

Processing Liquid Media, *M.*	Water
Laser power, *P* (W)	3 W, 9 W, 15 W, 21 W
laser energy density	5.1 J/cm^2^, 15.3 J/cm^2^, 25.5 J/cm^2^, 35.7 J/cm^2^
Angle, *θ* (°)	0°, 15°, 30°, 45°, 60°
Pulse frequency, *f* (kHz)	30 kHz
Number of Scan passes, *N*	500 (constant)
Laser Wavelength, *λ* (nm)	1064 nm
Laser Spot Diameter	50 μm
Liquid thickness, *l* (mm)	1.0
Pulse Width	100 ns
Overlap Ratio	66.7%

## Data Availability

The raw data supporting the conclusions of this article will be made available by the authors on request.
